# Fibrinogen and a Triad of Thrombosis, Inflammation, and the Renin-Angiotensin System in Premature Coronary Artery Disease in Women: A New Insight into Sex-Related Differences in the Pathogenesis of the Disease

**DOI:** 10.3390/biom11071036

**Published:** 2021-07-15

**Authors:** Karolina E. Kryczka, Mariusz Kruk, Marcin Demkow, Barbara Lubiszewska

**Affiliations:** Department of Coronary and Structural Heart Diseases, National Institute of Cardiology, 04-628 Warsaw, Poland; mkruk@ikard.pl (M.K.); mdemkow@ikard.pl (M.D.)

**Keywords:** premature coronary artery disease, women, fibrinogen, atherosclerosis, inflammation, sex differences in CAD, the RA system, I/D polymorphism of the *ACE* gene

## Abstract

Coronary artery disease (CAD) is the leading cause of morbidity and mortality in women worldwide. Its social impact in the case of premature CAD is particularly devastating. Many differences in the presentation of the disease in women as compared to men, including atypical symptoms, microvascular involvement, and differences in pathology of plaque formation or progression, make CAD diagnosis in women a challenge. The contribution of different risk factors, such as smoking, diabetes, hyperlipidemia, or obesity, may vary between women and men. Certain pathological pathways may have different sex-related magnitudes on CAD formation and progression. In spite of the already known differences, we lack sufficiently powered studies, both clinical and experimental, that assess the multipathogenic differences in CAD formation and progression related to sex in different age periods. A growing quantity of data that are presented in this article suggest that thrombosis with fibrinogen is of more concern in the case of premature CAD in women than are other coagulation factors, such as factors VII and VIII, tissue-type plasminogen activator, and plasminogen inhibitor-1. The rise in fibrinogen levels in inflammation is mainly affected by interleukin-6 (IL-6). The renin–angiotensin (RA) system affects the inflammatory process by increasing the IL-6 level. Unlike in men, in young women, the hypertensive arm of the RA system is naturally downregulated by estrogens. At the same time, estrogens promote the fibrinolytic path of the RA system. In young women, the promoted fibrinolytic process upregulates IL-6 release from leukocytes via fibrin degradation products. Moreover, fibrinogen, whose higher levels are observed in women, increases IL-6 synthesis and exacerbates inflammation, contributing to CAD. Therefore, the synergistic interplay between thrombosis, inflammation, and the RA system appears to have a more significant influence on the underlying CAD atherosclerotic plaque formation in young women than in men. This issue is further discussed in this review. Fibrinogen is the biomolecule that is central to these three pathways. In this review, fibrinogen is shown as the biomolecule that possesses a different impact on CAD formation, progression, and destabilization in women to that observed in men, being more pathogenic in women at the early stages of the disease than in men. Fibrinogen is a three-chain glycoprotein involved in thrombosis. Although the role of thrombosis is of great magnitude in acute coronary events, fibrinogen also induces atherosclerosis formation by accumulating in the arterial wall and enabling low-density lipoprotein cholesterol aggregation. Its level rises during inflammation and is associated with most cardiovascular risk factors, particularly smoking and diabetes. It was noted that fibrinogen levels were higher in women than in men as well as in the case of premature CAD in women. The causes of this phenomenon are not well understood. The higher fibrinogen levels were found to be associated with a greater extent of coronary atherosclerosis in women with CAD but not in men. Moreover, the lysability of a fibrin clot, which is dependent on fibrinogen properties, was reduced in women with subclinical CAD compared to men at the same stage of the disease, as well as in comparison to women without coronary artery atherosclerosis. These findings suggest that the magnitude of the pathological pathways contributing to premature CAD differs in women and men, and they are discussed in this review. While many gaps in both experimental and clinical studies on sex-related differences in premature CAD exist, further studies on pathological pathways are needed.

## 1. Introduction

Cardiovascular diseases are the leading cause of death worldwide, with coronary artery disease (CAD) being the most common [1]. At the same time, the annual cardiovascular mortality rate is increasing worldwide and is predicted to reach 23.6 million in 2030 [1]. Although CAD is thought to be more prevalent in men than in women, the incidence of CAD in young women is rising, and it remains the leading cause of morbidity and mortality in this important social group worldwide [2,3]. 

The presentation of the disease differs in women vs. men, which leads to CAD being underdiagnosed in women [2]. Among these differences, the atypical symptoms, microvascular involvement, the variable impact of cardiovascular risk factors such as smoking or diabetes, and CAD morbidity occurring approximately 10 years later than in men are of particular concern [2,3,4,5,6,7,8,9,10,11]. According to the SCORE study, age may become a significant cardiovascular risk factor at a cutoff in women above 55 years of age and in men above 45 years of age [12]. Therefore, it seems reasonable to differently define premature CAD as occurring before or at the age of 55 in women and before or at the age of 45 in men [12]. 

In younger patients and women, non-classical risk factors, such as genetic mutations, psychosocial factors, and anatomical characteristics, may play a relatively more important role in the pathogenesis of CAD [13,14], which calls for a careful insight into the pathogenesis of atherosclerosis in young women [15]. 

In this review, firstly, we present the interplay between coagulation, inflammation, and the renin–angiotensin (RA) system and its altered role in atherosclerosis formation in premature CAD in women vs. men.

Thrombosis is the one of processes that influences atherosclerosis differently in younger women and men of all ages. Thrombosis and clot structure have a substantial role in CAD and acute coronary events in both women and men [16]. However, in the VIRGO study that encompassed patients with premature CAD at the early stages of the disease with non-obstructive coronary arteries, a hypercoagulable state was found only in women and not in men, suggesting a more significant role of thrombosis at the early stages of the disease in women than in men [17].

Inflammation is another pathway that plays a substantial role in atherosclerosis formation, as well as the modulation of the thrombosis process. In the case of some markers of inflammation, such as urine 11-dehydrothromboxane B2, the oxLDL/β2GPI complex, or oxidized LDL, no significant differences between women and men were found [18,19]. Some studies indicated a higher mean concentration of C-reactive protein (CRP) in women than in men [20,21]. The differences are mainly observed in premenopausal women, as estrogens increase levels of CRP, IL-6, IL-1β, and tumor necrosis factor (TNF) [21]. However, no association of CRP levels with coronary artery calcium (CAC) was found in healthy women [20]. This probably results from other cardiovascular protective mechanisms influenced by estrogens [21]. The positive association of higher CRP levels with CAC was only found in the case of coexisting other cardiovascular risks: diabetes in women and obesity in men [20].

In women, estrogens decrease the activity of the main vasoconstrictive arm of the RA system and activate a cardioprotective, vasodilating part [22]. The function of the RA system, which also affects thrombosis and endothelial inflammation and plays a substantial role in cardiovascular homeostasis, including atherosclerosis formation, is known to be differently regulated in women and in men [22,23]. Taking these differences into account, the relationships and modulation between thrombosis, inflammation, and the RA system may have a different impact on the pathogenesis of atherosclerosis in young women, and this is discussed in the present review [15]. 

**Figure 1:**
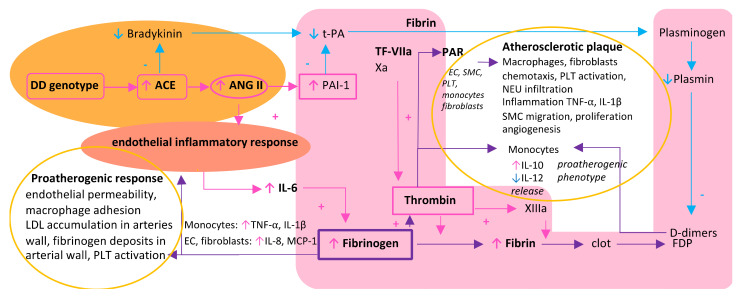
The role of coagulation, the renin–angiotensin system, and inflammation in atherosclerotic plaque formation. Orange background encapsulates the renin-angiotensin system; pink background embraces thrombotic processes; yellow rings encapsulate atherosclerotic processes. Abbreviations: Xa, activated coagulation factor X; XIIIa, activated coagulation factor XIII; ACE, angiotensin-converting enzyme; +, addition; Ang II, angiotensin II; DD, dominant homozygote of the insertion/deletion polymorphism of the angiotensin-converting enzyme gene; ↓, decrease; EC, endothelial cell; FDPs, fibrinogen degradation products; IL, interleukin; ↑, increase; −, inhibition; LDL, low-density lipoprotein cholesterol; MCP, monocyte chemoattractant protein; NEU, neutrophil; PAI-1, plasminogen activator inhibitor 1; PAR, protease-activated receptor; PLT, platelet; tPA, tissue plasminogen activator; SMC, smooth muscle cell; TF-VIIa, tissue factor-activated coagulation factor VII complex; TNF, tumor necrosis factor.

Clinically, it is difficult to measure the result of the interactions of these three pathways. One of the biomolecules that may reflect the magnitude of these interactions is fibrinogen: a main thrombotic factor, an acute-phase protein, the RA system effector, and a factor directly inducing atherosclerosis formation [24] (Figure 1). In this review, clinical data from studies on fibrinogen’s role in CAD are presented, and its different meaning for women and men, especially in the case of premature CAD, is discussed. 

The rising evidence supports the thesis that fibrinogen, the multifunctional protein, is more responsible for CAD formation and progression in young women than in men, which is further discussed in this review [17,24,25,26,27,28,29,30,31]. Mentioned differences in premature CAD presentation, progression, and outcome in women and men indicate that the importance of different pathways and factors may vary between both sexes, calling for further investigation. 

## 2. A Triad of Thrombosis, Inflammation, and Renin–Angiotensin System in the Vicious Circle of Atherosclerosis Formation—Differences between Women and Men

Thrombosis, inflammation, and the RA system are known to contribute to the etiology of atherosclerosis in many different aspects [32,33,34]. What is even more important is that these three processes may influence one another, modulating their pathological impact. 

Thrombosis is an important pathological pathway in CAD in both women and men. Thrombosis activation may be affected by inflammation mainly by interleukin (IL)-6, which stimulates the expression of tissue factor (TF) on the surface of circulating monocytes (e.g., during sepsis) or macrophages in atherosclerotic lesions [32]. In the case of CAD, TF has direct contact with circulating blood after plaque rupture and induces thrombosis by forming active complexes with factor VIIa (TF-FVIIa). IL-6 also enhances thrombosis by increasing fibrinogen levels [35]. These facts are consistent with the observation that gene polymorphisms of IL-6 have been found to increase cardiovascular risk [35]. Increased synthesis of IL-6, which mediates fibrinogen synthesis in the liver, is induced by the inflammatory response of endothelium playing a pivotal role in the pathophysiology of atherosclerosis [35,36]. This endothelial inflammation may be caused by increased activity of the RA system. The RA system is widely associated with atherosclerosis not only due to its influence on blood pressure but also inflammation and thrombosis [33,34] (Figure 1). Increased activity of the RA system decreases plasmin formation and clot lysis. Moreover, the RA system may increase IL-6 levels by enhancing the endothelial inflammation process (Figure 1). 

**Figure 2:**
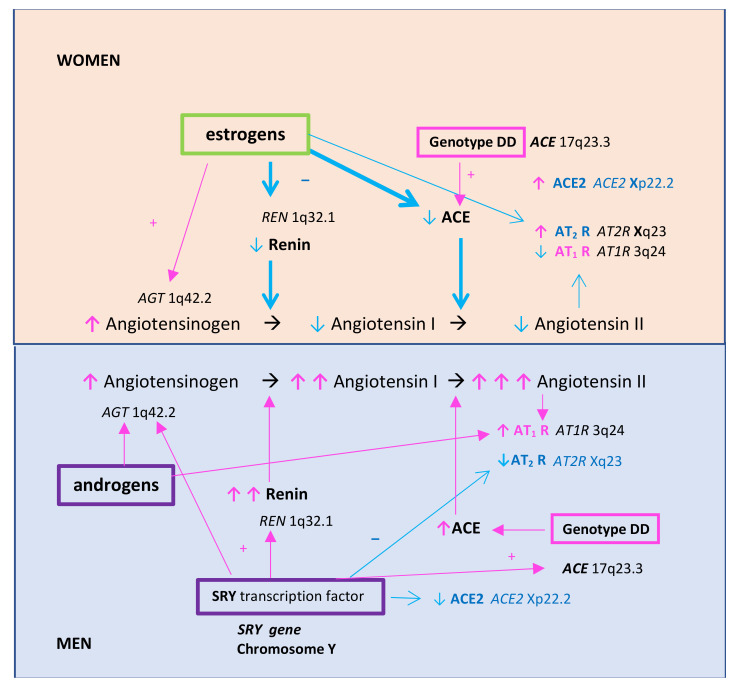
Differences in the regulation of the renin–angiotensin system in women vs. men. Abbreviations: ACE, angiotensin-converting enzyme; +, addition; *AGT*, angiotensinogen gene; AT_1_R, angiotensin II receptor type 1; AT_2_R, angiotensin II receptor type 2; DD, dominant homozygote of the insertion/deletion polymorphism of the angiotensin-converting enzyme gene; ↓, decrease; ↑, increase; −, inhibition; *REN*, renin gene.

The overall activity of the RA system was found to vary according to sex, with lower activity in women than in men [22] (Figure 2). 

One of the causes of this difference is the protective role of estrogens in young women. Estrogens regulate the expression of genes that encode the enzymes of the RA system, resulting in increased synthesis of angiotensinogen but a decreased synthesis of renin and angiotensin-converting enzyme (ACE) [22,37]. Estrogens also act protectively by decreasing the expression of the angiotensin II receptor type 1 (AT_1_R) that mediates the hypertensive effect of angiotensin II and increasing the expression of the angiotensin II receptor type 2 (AT_2_R), which is a part of the depressing arm of the RA system [22]. The levels of ACE2, the main enzyme of the diastolic pathway of the RA system encoded by the gene localized on chromosome X, are twice as high in women than those found in men [22]. 

Testosterone stimulates the synthesis of angiotensinogen and AT_1_R [22]. The hypertensive and proinflammatory RA system pathway in men is also stimulated by the transcription factor SRY encoded by the *SRY* gene localized on chromosome Y. SRY increases the synthesis of angiotensinogen, renin, and ACE and decreases ACE2 and AT_2_R synthesis [22] (Figure 2).

These differences cause the baseline activity of the RA system to be lower in premenopausal women than in men. Therefore, in young women, additional risk factors that influence the activity of the RA system, such as insertion/deletion (I/D) polymorphism of the *ACE* gene, may be of particular concern. A crucial enzyme responsible for the activity of the RA system is ACE, a zinc metalloproteinase that cleaves angiotensinogen II into active angiotensin II [38]. The level of ACE varies according to genotype. It is the highest in patients with the DD genotype of the *ACE* gene, intermediate in ID heterozygotes, and the lowest in homozygotes II with no differences according to sex [38]. Some data suggest that the DD genotype or other factors acting synergistically with the RA system, e.g., higher fibrinogen levels, may be particularly important in the case of premature CAD in women [15]. As shown in Figure 2, the DD genotype is in practice only one strong factor that increases the activity of the RA system in premenopausal women; therefore, its role in premature CAD in women is of greater concern than in men. In men, apart from the DD genotype, other factors strongly increase the activity of the RA system, namely, the SRY transcription factor and androgens (Figure 2) [22]. Higher fibrinogen levels may also result from some mutations in fibrinogen polypeptide chain genes that lead to increased fibrinogen synthesis [39]. It was reported that women hospitalized for different conditions possessed clustered mutations in many more genes than those in men [40]. Therefore, premature CAD in women may be the result of several coexisting mutations in such genes as the *ACE* and fibrinogen genes [15]. Unfortunately, no studies comparing the frequency of mutations in fibrinogen genes in women and men with CAD have been conducted to date [39].

## 3. Fibrinogen as a Resultant and the Central Biomolecule in a Triad of Thrombosis, Inflammation, and Renin–Angiotensin System 

Fibrinogen (FG) is a 340 kDa glycoprotein that consists of three pairs of polypeptides chains (Aα/Bβ/Gδ) joined by sulfate bridges [16]. The polypeptide chains of fibrinogen are synthesized in hepatocytes and encoded by three different genes localized on chromosome 4 [16,39]. The regulation of the synthesis of the β-FG chain is crucial for the fibrinogen formation process. Therefore, mutations in the β-FG gene are mainly responsible for alterations in plasma fibrinogen levels [41]. Polymorphisms of β-FG are associated with increased fibrinogen levels, especially in smokers [39,42], while alternative fibrinogen variants, such as gamma prime, may lead to the formation of a thrombus that is resistant to lysis observed in patients with myocardial infarction [43]. 

### 3.1. Fibrinogen and Its Central Role in Thrombosis

During the coagulation process, fibrinogen is transformed into fibrin by thrombin, which cleaves A and B chains and enables fibrin polymerization and clot formation [16]. 

Previous studies have shown that among the factors involved in thrombogenesis and cardiovascular diseases (including factor VII, factor VIII, tissue-type plasminogen activator, and plasminogen inhibitor-1), fibrinogen was found to exert the highest impact on cardiovascular risk [44]. 

Fibrinogen can bind to plasminogen receptors disabling the plasminogen-induced thrombolysis and leading to decreased clot lysability [16,45,46]. This property of fibrinogen is of great concern in the case of higher fibrinogen plasma levels in women than in men [24].

As fibrinogen is a major coagulation factor, it is involved in increasing blood viscosity, enhancing coagulation, platelet aggregation, and clot formation [24]. Increased blood viscosity was found to increase cardiovascular risk and is caused by both the large size of fibrinogen and fibrinogen-induced platelet aggregation [47]. 

Fibrinogen levels were found to be the strongest determinant of activity of another important component of the coagulation cascade: thrombin. Thrombin is an enzyme that transforms fibrinogen into fibrin and activates platelets [25]. It is generated from prothrombin as a result of a cascade induced by the exposition of the sub-endothelial tissue factor (TF). When exposed to circulating blood, TF forms a complex with activated factor VII. Thrombin also triggers a positive feedback loop, and via activation of coagulation factors V, VIII, and XI, it induces further thrombin generation and fibrin formation [25]. 

A study conducted with a group of 134 healthy individuals assessed the three main parameters of the thrombin generation curve: the lag time (the initiation phase of the curve), the endogenous thrombin potential (ETP; the area under the curve representing the total amount of generated thrombin), and the peak time (representing the propagation phase). Women (*n* = 71) were characterized by an increased plasma thrombogenicity, defined by higher thrombin generation parameters, than that observed in men (*n* = 63) [25]. Further analyses revealed that among other thrombotic factors, fibrinogen levels were the main determinant of all three thrombin generation parameters [25]. Women, with higher fibrinogen levels, seem to be more prone to thrombosis, even in the case of a low TF concentration environment [25,48,49]. However, taking into account the low number of patients studied (*n* = 134), this phenomenon of sex differences in thrombin formation requires confirmation in further studies [25].

### 3.2. Fibrinogen as Both an Effector and Stimulator of Inflammatory Reaction

As previously mentioned, fibrinogen levels are altered by inflammatory processes. Fibrinogen synthesis is regulated by acute-phase proteins, mainly by IL-6, which induces its synthesis in the liver, while IL-1β and tumor necrosis factor-alpha (TNF-α) suppress its synthesis [35,36]. In return, fibrinogen and fibrin can promote an inflammatory response by inducing the exposition of proinflammatory cytokines on monocytes (TNF- α and IL-1β) as well as chemokines, such as IL-8 and monocyte chemoattractant protein-1 (MCP-1), on endothelium and fibroblasts [35,36,50]. This process increases monocyte diapedesis and accumulation in artery walls, leading to atherosclerotic plaque formation [35,36,50]. Fibrinogen also enhances inflammation by stimulating platelets via glycoprotein (GP) IIb/IIIa receptors. Activated platelets produce pro-inflammatory cytokines IL-1beta and CD40 ligand, which are involved in the development of atherosclerotic lesions [32].

### 3.3. Fibrinogen-Induced Atherosclerosis Formation 

Fibrinogen is not only a prothrombotic and proinflammatory factor or a marker of an ongoing acute process associated with inflammation but also directly induces atherosclerosis formation [24]. 

By binding to intercellular adhesion molecule-1 (ICAM-1) on endothelial cells, fibrinogen increases the expression of ICAM-1, which also binds leukocytes, macrophages, and platelets [51]. Additionally, fibrinogen binds to endothelium and induces the secretion of vasoactive substances that increase endothelium permeability [52]. This process triggers atherosclerosis by enabling the formation of fibrinogen aggregation within arterial walls as well as the infiltration of macrophages, which are the precursors of foam cells [24,53]. Fibrinogen deposits adsorb low-density lipoprotein (LDL) cholesterol and enable atherosclerotic plaque formation [24,53]. Additionally, fibrinogen may contribute to atherosclerotic plaque formation by mediating neutrophil adhesion to activated platelets attached to the injured arterial wall [54].

## 4. Distinct Clinical Impact of Fibrinogen on Coronary Artery Disease in Women and Men

### 4.1. Fibrinogen as an Independent Cardiovascular Risk Factor 

Patients with cardiovascular diseases were found to have higher fibrinogen levels that were also associated with the severity of atherosclerosis in both men and women [55,56]. A meta-analysis of 31 prospective studies with a total of 154,211 participants revealed that the age- and sex-adjusted hazard ratios (HRs) ranging between 2–3 per 1 g/L increase in the fibrinogen level for CAD (HR = 2.42), stroke (HR = 2.06), other vascular mortality (HR = 2.76), nonvascular mortality (HR = 2.76) [57]. The HR was slightly diminished for CAD and stroke after adjusting for traditional risk factors and equaled approximately 1.8, but it did not change after adjusting for C-reactive protein [57]. No sex-related differences were noted; however, in this analysis, women represented only 13.3% of analyzed participants [57]. On the other hand, some data indicate that the fibrinogen level in the general population was higher in women than in men [24]. As an increased fibrinogen level is recognized as a risk factor for CAD, this may indicate a greater influence of fibrinogen on cardiovascular diseases in women than in men [24,58]. In another study that included 516 participants from the general population (262 men and 254 women) at a mean age of 46.5 ± 10.7 years, among nonsmokers, fibrinogen levels were higher in women (290 mg/dL than in men (267 mg/dL) [59]. The fibrinogen level was found to be especially high in both men (293 mg/dL) and women (292 mg/dL) who smoked and increased with age in men [59]. Almost every traditional risk factor promoting the occurrence of CAD, including smoking, hypertension, diabetes, hyperlipidemia, obesity, age, and menopause, was found to be related to increased fibrinogen levels in both women and men [24]. At the same time, higher fibrinogen levels were found to increase cardiovascular risk independently of other traditional risk factors [57,60,61]. 

Fibrinogen also may be crucial for initiation of atherosclerosis plaque formation, as it was shown that even in patients with high LDL cholesterol, the cardiovascular risk was not increased if the fibrinogen level was low [62]. This suggests that fibrinogen deposits in artery walls may be necessary to adsorb LDL particles and trigger atherosclerotic plaque formation and growth.

### 4.2. Relationship between Fibrinogen and CAD in Women and Men

A different impact of fibrinogen on cardiovascular risk in women and men was discussed in the sub-analysis of the 12-year follow-up of the Framingham Heart Study. The follow-up encompassed 1315 subjects (aged 47–79 years old) with measured baseline fibrinogen. In the study group, 147 women and 165 men experienced a cardiovascular event [55]. The mean fibrinogen level at baseline equaled 291 mg/dL and rose by approximately 10 mg/dL with every 10-year increase in age. Moreover, baseline fibrinogen levels were higher in women than in men [55]. In men, fibrinogen levels were significantly associated with both coronary heart disease and stroke. In women, however, the association was significant only for coronary heart disease, and the effect was more pronounced in the younger group of 47–59 years of age [55].

Recently, a large study of 2690 patients undergoing angiography due to acute coronary syndrome (ACS) confirmed that women under 55 years of age have a five times higher risk of myocardial infarction with nonobstructive coronary arteries (MINOCA) compared to men of the same age. At the same time, contrary to women with coronary artery stenoses ≥50%, women with MINOCA were characterized by hypercoagulable states [17]. Of note, the hypercoagulable state was not observed in men with ACS [17]. These observations indicate the different role of fibrinogen in CAD in women than in men, especially at the early stage of the disease. 

One study showed that in patients who died from CAD, coronary thrombosis occurred more frequently in men than in women (53% vs. 46%) [26]. This observation indicates that while in women fibrinogen seems to be more important at the beginning of atherosclerosis formation, in men, fibrinogen may be a more important cardiovascular risk factor at a more advanced stage of CAD, especially when ACS is observed.

Most of the current knowledge on fibrinogen’s role in CAD originates from studies conducted in the 1970s–1990s. The studies revealed that fibrinogen levels were extremely high in men with CAD who smoked [62,63,64,65]. However, in these studies, women were the minority. The PROCAM study, which encompassed 2116 men between 40 and 65 years of age (mean 48.9 years) with no history of myocardial infarction or stroke, revealed that plasma fibrinogen concentrations over 300 mg/dL were associated with an approximately two-fold increase in cardiovascular risk in a six-year follow-up [62]. In a metanalysis, which encompassed 52 studies, the fibrinogen was also found to predict future events in people with no history of cardiovascular disease and intermediate cardiovascular risk [60]. In this large population of 246,669 participants, assessing fibrinogen levels or CRP levels resulted in the prevention of one additional cardiovascular event in 10-year observation for every 400 to 500 people screened [60]. In another smaller study that aimed to discover proteins associated with cardiovascular events in low cardiovascular participants, fibrinogen was significantly associated with cardiovascular events during the first 3 years of follow-up in a group of 50 participants [66]. However, in the extended population of 151 participants of older age and with relatively low cardiovascular risk, there was no association found between fibrinogen and cardiovascular events during 5-year follow-up [65]. The results of this study also support the thesis that fibrinogen’s role in CAD may be more important in younger patients. 

It was also shown that other coagulation factors may contribute to increased cardiovascular risk. In the Northwick Park Heart Study, deaths caused by ischemic heart disease in a group of 1511 men aged 40–64 years at baseline were most frequent in patients with elevated values of factor VII activity (VIIc), factor VIII activity (VIIIc), and fibrinogen [64]. In the majority of patients with the fatal outcome of coronary heart disease, a clustering of elevated values of two or three thrombotic factors, i.e., factor VIIc, factor VIIIc, and fibrinogen, was observed [64].

In the second analysis of this group, which included the fatal and nonfatal ischemic heart events during a mean follow-up of 10 years, the standardized regression effects (SREs) that indicated an increased risk of an event were the highest for fibrinogen, followed by factor VII and cholesterol (SRE 1.5, 1.24, and 1.35, respectively) [65]. The association was especially strong for fibrinogen in the first five years of follow-up, with SREs of 1.84, 1.62, and 1.43 for fibrinogen, factor VII, and cholesterol, respectively. The risk of fatal and nonfatal events gradually increased and was the highest in the third tertile of fibrinogen levels (over 3.19 g/L) [65]. Among other studied thrombotic factors (which included platelet count; platelet adhesiveness to glass beds; fibrinolytic activity; biological activity assays of factors V, VII, and VIII; and antithrombin III), only the activity of factor VII was associated with a fatal outcome of ischemic heart disease [67]. Within the first five years of the follow-up, a synergistic effect of fibrinogen and factor VII was observed. The frequency of ischemic heart events rose from 2.5% for those with neither factor VII nor fibrinogen in the third tertile to 4% for one risk factor value in the third tertile and up to 10.4% for both values of fibrinogen and factor VII being in the third tertile [65].

### 4.3. Smoking as a Modifier of Fibrinogen Impact on CAD

Smoking seems to be one of the main modifiers of fibrinogen’s impact on cardiovascular risk. At the entry of the study, the mean fibrinogen levels were found to be especially elevated in smoking men with ischemic disease events (3.23 g/L) compared to nonsmokers without a cardiovascular event (2.75 g/L). However, in the 1970s–1990s, women were the minority in medical trials and smoking was less common in women than in men [65]. These two facts precluded the adequate comparing of fibrinogen’s impact on the cardiovascular risk between women and men. In the 1970s, the frequency of smoking in women of 35–54 years of age in the general population of United States equaled approximately 37.5%, and in women of 55–64 years of age, it was approximately 30%. For men, the respective values were 48% and 38% [68]. In the whole cohort of the Northwick Park Heart Study, which encompassed 941 women (30% of the study group), the frequency of smoking was 37% in women at a mean age of 48.3 ± 14.5 and 48% in men at a mean age of 48.1 ± 18.1 [65]. The 29-years of follow-up of the Northwick Park Heart Study showed that in the studied group of women, neither smoking nor fibrinogen was associated with an increased risk of cardiovascular death [67]. However, no analysis was provided according to premature CAD. Additionally, today, despite decreasing smoking rates in the general population, smoking is reported in the majority of women with premature CAD (approximately 60–70%) and is almost as frequent as in men [23,69]. Of note, in the 2000s the smoking frequency in the United States in the younger age group of 35–54 was approximately 21% for women and 25% for men, and in the elder age group of 55–64, it was approximately 16% for women and 21% for men [68].

Despite data from clinical studies presented above, the magnitude of fibrinogen cardiovascular risk seems to be underappreciated in cardiovascular guidelines and everyday clinical practice [70].

Although some studies did not analyze the association of fibrinogen levels with CAD and in other studies women were underrepresented or the age groups were not distinguished, some of the presented data suggest that the influence of fibrinogen may be especially important in the case of premature CAD in women and at an early stage of the disease.

### 4.4. Fibrinogen and Sex Differences in Atherosclerotic Plaque Morphology and Clot Lysability 

Recently, some studies suggested different fibrinogen impacts on plaque morphology and clot lysability in women at the early stage of CAD compared to those in men. 

In the study that encompassed 71 women (35 at the age of 55 and 36 at the age of 65) and 67 men (43 at the age of 55 and 24 at the age of 65), higher fibrinogen levels were observed in women with plaque volume > 0 mm^3^ in computed tomography coronary angiography than in men at the same stage of the disease, as well as in men with no atherosclerosis (10.1 μmol/L vs. 9.2 μmol/L, *p* < 0.05 and vs. 9.0 μmol/L, *p* < 0.05, respectively). Higher fibrinogen levels were found to be associated with lower clot lysability (r = −0.46). Women at an early subclinical stage of coronary atherosclerosis, with a total plaque volume > 0 mm^3^, had significantly lower fibrin clot lysability than did men at the same stage of the disease, as well as women without coronary artery atherosclerosis (39.3% vs. 50.9%, *p =* 0.06 and vs. 53.7%, *p* = 0.02, respectively) [27]. Moreover, fibrinogen levels correlated with all the vulnerable plaque features in women but not in men, i.e., low attenuation, spotty calcification, and vascular remodeling (*r* = 0.42–0.57) [27]. These findings suggest that the main mechanism of atherosclerosis formation at the early stages may be different in women and men. Recently, these pathological observations found confirmation in a clinical study that recruited 339 subjects who had plaques characterized in coronary computed tomography angiography [31]. Higher fibrinogen levels were associated with the presence of non-calcified plaques or mix plaques that are prone to rupture in women but not in men (OR: 3.677, *p* < 0.01) [31].

In another study that encompassed 442 patients (359 men, mean age of 48 ± 10 years; and 83 women, mean age of 50 ± 11 years) with sudden cardiac death, it was shown that the plaque morphology varied between women and men [26]. Plaque erosion was the leading cause of cardiac events in women but not in men (58% vs. 24%) [26]. Plaque rupture was more frequent in men than in women (71% vs. 33%). 

#### 4.4.1. The Role of Estrogens

When women were assessed according to age groups, in younger women aged under 50 years, plaque erosion was the leading cause of cardiac events as compared to older women (84% vs. 32%), while in women over 50 years of age, plaque rupture became the main mechanism as compared to younger women (53% vs. 16%) [26]. These data are consistent with the observations that stable atherosclerotic plaques in younger women aged under 50 years had thicker fibrotic caps than in older women and, therefore, were less prone to rupture. The mean proportion of plaques with a lipid core; the number of vulnerable, prone-to-rupture plaques; and the mean calcification score were lower in the younger group of women than in older women over 50 years of age [26,28]. 

The increased vulnerability of atherosclerotic plaques in elder women was pronounced even when compared with men. A study that encompassed 416 symptomatic elder patients (mean age of 61) with an intermediate to high risk of CAD showed that elder women (*n* = 148, mean age of 62) had more non-calcified, prone to rupture plaques (40% vs. 28%, *p* < 0.001) in coronary arteries than did men (*n* = 268, mean age of 60) [29]. Therefore, it is postulated that estrogens contribute to more stable plaque morphology and protect atherosclerotic plaques from rupture but not from erosion. The differences in plaque morphology and evolution may be a result of estrogens’ effect on reducing inflammation, which is the main trigger of plaque rupture [28]. 

On the other hand, the administration of exogenous female hormones was found to be associated with an increased risk of thrombosis [71]. It was shown that the use of oral contraceptives (OCs) in premenopausal women reduced levels of anticoagulation proteins, such as tissue factor pathway inhibitor (TFPI), activated protein C, and antithrombin III, which diminish thrombin formation [72,73]. OCs increased levels of factor VII and fibrinogen [74]. 

Hormone replacement therapy (HRT) when administered in early postmenopause decreases the risk of cardiovascular diseases [75]. However, the introduction of HRT in the later postmenopausal period increases the risk of cardiovascular diseases in women [75]. This is plausibly caused by already irreversibly increased stiffness of artery walls mainly caused by chronic estrogen deficiency. In early postmenopause, optimally during the first three years, arterial stiffness may be attenuated by HRT, reducing cardiovascular risk [75]. The increased cardiovascular risk associated with HRT in some women may be explained by increased activity of TF and decreased antithrombin III activity and TFPI, which inhibits coagulation at an early phase by blocking protease-cofactor complexes of TF–factor VIIa and prothrombinase [76]. These associations were found in a group of thirty women during treatment with micronized progesterone or medroxyprogesterone acetate with conjugated equine estrogen, showing that exogenous female hormones should be administered with caution [76]. 

#### 4.4.2. Stronger Impact of Smoking and Diabetes on Atherosclerosis in Women 

It was shown that smoking at least 20 cigarettes a day may increase cardiovascular risk in women by two times as much as in men (with a relative risk of six for women and three for men) [4,5]. The difference was even more pronounced in patients < 45 years of age at entry, with a relative risk of 7.1 for women and 2.3 for men [6]. Plaque erosion, which is more frequent in younger women, is mainly caused by vasospasm that leads to regional damage of the endothelium. An exposition of eroded plaque core rich in smooth-muscle cells and proteoglycans leads to local thrombosis with less severe reduction of arteries’ lumen than that observed after plaque rupture [22]. This may explain why smoking, which induces vasospasms and thrombosis, is a more important cardiovascular risk factor for women, especially at a younger age, than for men. 

Diabetes has also been found to be a stronger risk factor in women than in men [7,8]. A meta-analysis of 37 studies showed that type 2 diabetes increased the risk of fatal CAD by a relative risk of 3.5 for women and 2.1 for men [9].

In pathophysiological studies, eroded atherosclerotic plaques consisted of intimal thickening or fibroatheroma [26]. Diabetes may lead to increased intimal thickening, increased fibrinogen levels, and fibrinogen glycation proportional to the level of hyperglycemia [16]. Fibrinogen glycation leads to the production of a clot that is resistant to lysis [16,77]. It was also confirmed that women with type 2 diabetes have a more compact clot structure than do men [30,77]. 

These observations explain the seemingly stronger impact of diabetes and smoking on atherosclerosis formation in women (especially young women) than that in men. 

#### 4.4.3. Different Fibrinogen Levels and Plaque Composition in Women and Men 

As the composition of atherosclerotic plaques differs in men and mainly young women, the mechanism of thrombosis may also differ with a more significant role of fibrinogen level in younger women than in men. Indeed, coronary thrombus overlying the ruptured plaques, which more frequently occurs in men, consists mainly of activated platelets. On the other hand, the hyaluronan, secreted by vascular smooth muscle cells, is present in eroded plaques that are more frequent in younger women than in men. Hyaluronan, apart from promoting platelet aggregation, also triggers fibrin polymerization and smooth-muscle cell migration, which leads to plaque progression [26]. In the light of these observations, higher fibrinogen levels in younger women than those in men may be of more concern in the aspect of increased fibrin formation on eroded plaques and atherosclerosis evolution and progression.

## 5. Therapeutic Implications of Fibrinogen Lowering on Residual Vascular Risk

It is widely known that despite optimal management of cardiovascular diseases, some residual vascular risk remains [78]. The residual vascular risk is defined as the significant risk of macrovascular events and microvascular complications that persists after achieving recommended therapeutic goals in the management of LDL cholesterol, blood pressure, and glucose levels [79]. In the study that assessed 12,513 patients with high cardiovascular risk, reducing seven main modifiable cardiovascular risk factors (namely, hypercholesterolemia (the goal: LDL-C < 130 mg/dL; total cholesterol < 200 mg/dL), hypertension (<140/90 mmHg), diabetes (fasting glucose < 130 mg/dL, Hb1C < 7%), smoking (non-smoking), obesity (BMI < 30 kg/m^2^), physical inactivity (regular exercise) and unhealthy diet (healthy diet)) resulted in lasting residual risk of cardiovascular events at the level of 58% [80]. This percentage was estimated based on data showing that improvement in any of the mentioned risk factors during the first year of treatment resulted in a 6% reduction in cardiovascular events (cardiovascular deaths and hospitalization for a cardiovascular reason) in the next four-year follow-up [80]. Therefore, a need for further assessment of other modifiable cardiovascular risk factors exists. 

Despite the evidence that elevated fibrinogen is a significant cardiovascular risk factor, there is a lack of projects that would assess the cardiovascular risk reduction resulting from lowering the fibrinogen level. Therefore, widely accepted cardiovascular guidelines do not consider fibrinogen in risk assessment and recommend therapeutic options lowering fibrinogen levels [70]. 

Among oral drugs that can be used in long-term treatment, fibrates were reported to decrease elevated fibrinogen levels by approximately 40% [81] Apart from lowering the blood level of triglycerides, fibrates have pleiotropic effects that include lowering inflammatory cytokines, such as IL-6 and IL-1; reducing insulin resistance; and lowering uric acid concentration [82]. Micronized fenofibrate also affects clot structure, making it more prone to lysis [16]. Administrating fibrates may further reduce cardiovascular risk in patients with atherogenic dyslipidemia [83]. However, fibrates may also increase homocysteine levels that are associated with increased cardiovascular risk [83]. Therefore, studies assessing the overall influence of fibrates in certain cardiovascular risk groups are necessary. Moreover, ACE inhibitors were found to reduce fibrinogen levels, likely by decreasing the production of inflammation factors stimulated by angiotensin II [36]. Based on the Northwick Park Heart study, it can be estimated that a reduction in fibrinogen levels of 0.1 g/L would decrease cardiovascular risk by 15% [81]. This calculation appears to be quite encouraging but needs confirmation in the context of contemporary cardiovascular treatment, including ACE inhibitors, sartans, and statins. Apart from the above, many of the interventions that reduce the cardiovascular risk, such as changes in lifestyle, a bodyweight reduction, regular physical activity, smoking cessation, and a healthy diet, were also found to reduce fibrinogen levels, as does the relevant pharmacological treatment of cardiovascular diseases, including hypertension, hyperlipidemia, CAD, and diabetes [16,81]. 

As stated previously, smoking has been found to significantly increase fibrinogen levels, with the increase positively correlated with the number of cigarettes smoked [81]. The mechanism of this influence is not clear; however, it has been shown that smoking increases the inflammatory response of the endothelium [81]. This leads to an increase in inflammatory cytokine production, including IL-6, which stimulates fibrinogen synthesis in the liver. Smoking also induces the release of elastase from leukocytes, which enhances thrombolysis. In the next step, the fibrin degradation products stimulate leukocytes to release IL-6, which, in turn, promotes fibrinogen synthesis [81]. After smoking cessation, fibrinogen levels decrease to levels comparable with those of nonsmokers [56,83]. Obesity is another risk factor associated with increased fibrinogen levels. A decrease in body mass, associated with healthy diet intake, was found to lower fibrinogen levels [81]. Therefore, fibrates, especially micronized fenofibrate, may pose a therapeutic option in patients with preserved high fibrinogen levels despite optimal pharmacological treatment and a healthy lifestyle. However, overall cardiovascular risk reduction during such treatment needs to be elucidated. 

## 6. Conclusions

Thrombosis plays a significant role in the formation and clinical presentation of CAD in both women and men. It was shown that elevated levels of one of the central biomolecules, fibrinogen, in the coagulation process are closely associated with an increased risk of thrombosis contributing to CAD progression. Fibrinogen increased cardiovascular risk irrespective of traditional cardiovascular risk factors and on a comparable level. Moreover, elevated fibrinogen levels modified the risk of other factors, such as increased LDL or factor VII. 

Fibrinogen associations with increased risk of CAD were mainly reported in prospective cohort and observational studies, much of them without sub-analyses according to age and sex subgroups. However, some data indicate that the role of fibrinogen in CAD formation may differ between women and men. As women at a younger age are naturally protected by estrogens, which decrease the RA system’s activity and inflammation, additional risk factors are likely responsible for premature CAD, with fibrinogen being one of the most targetable.

Decreased thrombus lysability and a higher level of fibrinogen in women at an early asymptomatic stage of CAD indicate a significant role of fibrinogen in atherosclerotic plaque formation in women with premature CAD, especially at the first stage of the disease. Fibrinogen deposits in arterial walls trigger atherosclerotic plaque formation by activating the local inflammatory response, adsorbing LDL cholesterol, increasing monocyte chemotaxis, and causing smooth-muscle cell migration. Local micro-thrombosis on eroded plaques consisting of fibrin deposits is typical for younger women but not for men. Therefore, fibrinogen levels seem to be of main concern in the atherosclerotic plaque progression process in premature CAD in women. 

The above findings highlight the need for more studies and clinical trials that separately assess the impact of fibrinogen levels on cardiovascular risk in women and men according to age and other cardiovascular risk factors. Such trials could become the basis for new cardiovascular risk stratification scales. The next step should involve the assessment of the cardiovascular risk-reducing role of fibrates administrated on top of the currently recommended optimal medical treatment and healthy lifestyle. These actions would systemize current cardiovascular knowledge and risk assessment, likely translating it into improved patient care and outcomes.
